# UAVs Maneuver Decision-Making Method Based on Transfer Reinforcement Learning

**DOI:** 10.1155/2022/2399796

**Published:** 2022-11-14

**Authors:** Jindong Zhu, Xiaowei Fu, Zhe Qiao

**Affiliations:** ^1^School of Electronics and Information, Northwestern Polytechnical University, Xi'an 710072, China; ^2^AVIC Shenyang Aircraft Design & Research Institute, Shenyang 110035, China

## Abstract

Aiming at the 1vs1 confrontation problem in a complex environment where obstacles are randomly distributed, the DDPG (deep deterministic policy gradient) algorithm is used to design the maneuver decision-making method of UAVs. Traditional methods generally assume that all obstacles are known globally. In this paper, a UAV airborne lidar detection model is designed, which can effectively solve the problem of obstacle avoidance when facing a large number of unknown obstacles. On the basis of the designed model, the idea of transfer learning is used to transfer the strategy trained by one UAV in a simple task to a new similar task, and the strategy will be used to train the strategy of the other UAV. This method can improve the intelligence of the UAVs in both sides alternately and progressively. The simulation results show that the transfer learning method can speed up the training process and improve the training effect.

## 1. Introduction

In the battlefield, UAVs can play a role in reconnaissance, detection, target tracking, attack interception, damage assessment, and others [1]. UAVs can also be used to intercept the enemy UAV [2]. How both sides maneuver to achieve the corresponding task objectives has aroused the attention and research interest of military experts and a large number of scholars.

At present, many experts have proposed different algorithms to solve the maneuver decision-making problems in different situations. In the traditional method, the main algorithms are the differential game method [3], expert system method [4], and guidance law [5]. These methods have shown good effect on simple tasks, but they cannot be applied to complex battlefields where the environment is unknown, and it is difficult to obtain analytical solutions. Therefore, scholars try to apply intelligent algorithms to UAV attack and defense confrontation problems, including bionic modeling [6], fuzzy cybernetics [7], and swarm intelligence algorithms [8].

Deep reinforcement learning, as an artificial intelligence technology that combines neural networks and reinforcement learning, is a new type of a decision-making method, which has good application prospects for the research of UAV countermeasures. For the scenario of UAV swarms chasing enemy targets, the DDPG algorithm is used to train UAVs to pursue targets [9]. Aiming at the confrontation problem with multiple UAVs, the cooperative decision-making method of multiple UAVs based on the multiagent reinforcement learning algorithm is proposed [10]. An MPPO algorithm is proposed to solve the confrontation problem of a large-scale UAV swarm [11]. A hierarchical framework based on reinforcement learning and two kinds of motion planning strategies for the problem of chasing and escaping games in the presence of obstacles is presented [12]. Liu and Wang proposed an adversarial decision generation method based on the generative adversarial network for the confrontation between UAVs in a barrier-free environment [13]. Wen and Shi proposed an intelligent decision making method for multicoupled tasks of cluster UAV confrontation in complex environments [14]. Wang and Guo improved the reward function of the cluster UAV confrontation model and optimized the reward calculation method [15]. These works have verified the feasibility of applying deep reinforcement learning to the UAV confrontation problem. Most of the current research is carried out under the condition that the scene information is completely known, and the designed strategy is suitable for specific confrontation scenarios. If the scene becomes complicated, these studies may turn ineffective.

In this paper, to solve the problem of obstacle avoidance when facing a large number of unknown obstacles, a UAV airborne lidar detection model is designed, and a 1vs1 maneuver decision-making method based on the DDPG algorithm is proposed. To get a better training effect, three training methods are designed by the idea of transfer learning. The scenarios corresponding to these three training methods are interrelated, that is, gradually increasing the task difficulty and fixing the strategy of the other UAV when one UAV is trained so as to make the confrontation environment of the agent relatively stable. We can transfer the relevant experience gained during the interaction between the UAV and the environment into new training scenarios to improve the intelligence of the UAVs on both sides alternately and progressively. The experimental comparison between the transfer and nontransfer methods shows that the transfer reinforcement learning makes the two UAVs have their own intelligent strategies in a 1vs1 confrontation game. It also shows that the method can speed up the training process and improve the confrontation effect.

## 2. Problem Description and Modeling

### 2.1. 1vs1 Confrontation Problem

**Figure 1:**
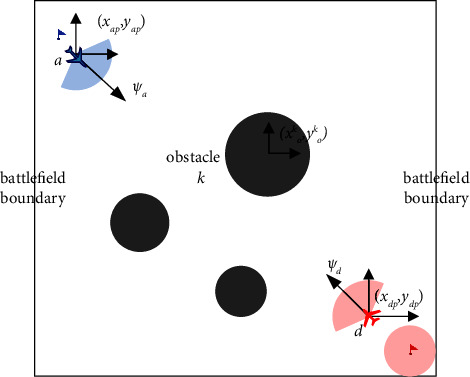
The scenario of 1vs1 attacking and defensive confrontation.

The scenario of 1vs1 confrontation can be described as that there are one blue UAV and one red UAV in a limited planar area, which are called the attack UAV and the defense UAV. The purpose of the attack UAV is to break through the interception of the defense UAV to reach the target area (light red area in the figure) from the initial position (blue flag). The purpose of the defense UAV is to intercept and destroy the attack UAV from the initial position (red flag). As shown in Figure 1, this paper assumes that circular obstacles (black areas) are distributed in the environment randomly. Only when the obstacles are within the detection range of the UAV's airborne radar, the UAV can obtain their positions.

In Figure 1, *a* and *d* represent the attack UAV and the defense UAV, respectively. *s*_*ip*_=(*x*_*ip*_, *y*_*ip*_)(*i*=*a*, *d*) represents the position coordinates of the UAVs. *s*_*id*_=*ψ*_*i*_(*i*=*a*, *d*) represents the heading angle of the UAVs. *R*_*a*_ and *R*_*d*_ represent the radar detection radius of the UAVs, respectively. (*x*_*tp*_, *y*_*tp*_) represents the position of the center point of the target area. *R*_*t*_ represents the effective radius of the target area. *s*_*op*_^*k*^=(*x*_*o*_^*k*^, *y*_*o*_^*k*^) represents the position of the *k*th obstacle center point. For the convenience of research, there is a battlefield boundary in the limited confrontation environment, and neither UAV can move out of the boundary.

It is assumed that the defense UAV can obtain the position and heading of the attack UAV in real time through the ground surveillance radar, and both sides carry lidar to detect obstacles and boundary of the local environment. It is also assumed that the attack UAV knows the position of the ground target area in advance.

### 2.2. Kinematics Model of UAVs

It is assumed that the UAVs fly in a two-dimensional plane. The kinematics equations of the UAVs are shown in formula:

(1) $$\begin{array}{c} \left\{\begin{array}{c} {\overset{˙}{x}}_{ip}=v_{i}\cos\psi_{i},\\ {\overset{˙}{y}}_{ip}=v_{i}\sin\psi_{i},\\ {\overset{˙}{v}}_{i}=a_{i}\text{,}\\ {\overset{˙}{\psi }}_{i}=\omega_{i}\text{,} \end{array}\right.\left(i=a,d\right)\text{,} \end{array}$$

where *v*_*i*_ represents the speed of the UAVs. *a*_*i*_ and *ω*_*i*_ represent the acceleration and angular velocity of the UAVs, respectively.

(2) $$\begin{array}{c} \left\{\begin{array}{c} x_{\min}\leq x_{ip}\leq x_{\max\;,}\\ y_{\min}\leq y_{ip}\leq y_{\max}\text{,}\\ 0\leq v_{i}\leq v_{i\;\max}\text{,}\\ 0\leq \psi_{i}<2\pi \text{,} \end{array}\right.\left(i=a,d\right)\text{,}\\ \left\{\begin{array}{c} -a_{i\;\max}\leq a_{i}\leq a_{i\;\max}\text{,}\\ -\omega_{i\;\max}\leq \omega_{i}\leq \omega_{i\;\max}\text{,} \end{array}\right.\left(i=a,d\right)\text{,} \end{array}$$

where *x*_min_, *x*_max_ and *y*_min_, *y*_max_ represent the boundary of the area. *v*_*i*max_ represents the upper limit of the UAV speed. *a*_*i*max_ represents the maximum value of the UAV acceleration. *ω*_*i*max_ represents the maximum value of the UAV angular velocity.

The current state of the UAV *i* is [*x*_*ip*_^*t*^, *y*_*ip*_^*t*^, *v*_*i*_^*t*^, *ψ*_*i*_^*t*^], and the state will change under the action of the acceleration *a*_*i*_ and angular velocity *ω*_*i*_. The state [*x*_*ip*_^*t*+1^, *y*_*ip*_^*t*+1^, *v*_*i*_^*t*+1^, *ψ*_*i*_^*t*+1^] at the next moment will be determined by the state transition equation as

(3) $$\begin{array}{c} \left\{\begin{array}{c} x_{ip}^{t+1}=x_{ip}^{t}+v_{i}\cdot \Delta T\cdot \cos\left(\psi_{i}^{t}+\omega_{i}\cdot \Delta T\right),\\ y_{ip}^{t+1}=y_{ip}^{t}+v_{i}\cdot \Delta T\cdot \sin\;\left(\psi_{i}^{t}+\omega_{i}\cdot \Delta T\right)\text{,}\\ v_{i}^{t+1}=v_{i}^{t}+a_{i}\cdot \Delta T\text{,}\\ \psi_{i}^{t+1}=\psi_{i}^{t}+\omega_{i}\cdot \Delta T\text{,} \end{array}\right\}\left(i=a,d\right)\text{.} \end{array}$$

### 2.3. Radar Detection Model

**Figure 2:**
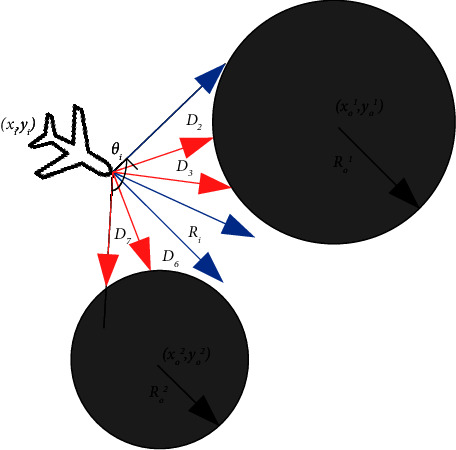
Obstacles detected by the UAV radar.

**Figure 3:**
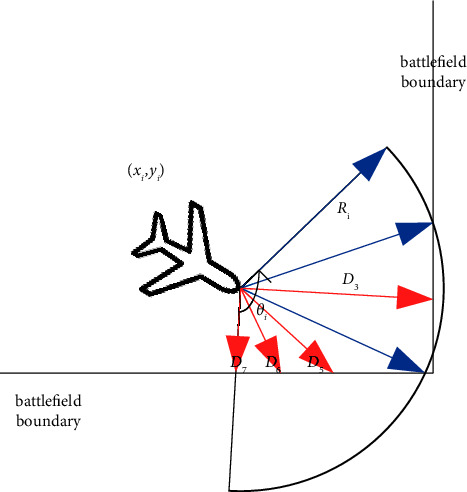
UAV radar detects the boundary of the battlefield.

It is assumed that both UAVs are equipped with lidar to detect the circular obstacles and enemy in the environment. As shown in Figures 2 and 3, the detection area of the UAVs is discretized into *m* state variables. In the figures, *R*_*i*_(*i*=*a*, *d*) represents the UAV radar detection radius. *θ*_*i*_(*i*=*a*, *d*) represents the detection angle range. *R*_*o*_^*k*^(*k*=1, ⋯, *N*_*k*_) represents the radius of the circular obstacle, where *N*_*k*_ represents the number of obstacles with different radius sizes. (*x*_*o*_^*k*^, *y*_*o*_^*k*^)(*k*=1, ⋯, *N*_*o*_) represents the position of the obstacles, where *N*_*o*_ represents the total number of obstacles.

As shown in Figures 2 and 3, in order to better represent the detection state of the radar, the detection angle range of the UAV radar is discretized into *l* (*l* = 7) directions at equal intervals. In the figure, it is represented by 7 rays, and the length of each ray is *D*_*n*_ (*n* = 1,…, l). The length of the blue ray is the maximum detection radius of the UAV radar, and the length of the red ray is the relative distance between the UAV and the obstacle or boundary detected in the corresponding direction. *x*_*io*_^*n*^(*i*=*a*, *d*)(*n*=1, ⋯, *m*) represents the ratio of *D*_*n*_ to the UAV radar maximum detection radius. If the ratio is closer to 1, it indicates that the UAV is farther from the obstacle or boundary in this direction. Otherwise, it indicates that the UAV is closer to the obstacle or boundary in this direction.

## 3. 1vs1 Confrontation Maneuver Decision-Making Method Based on Reinforcement Learning

In this paper, the reinforcement learning algorithm of DDPG is used to study the 1vs1 confrontation scenarios. Before using this algorithm, it is necessary to define the state space, action space, and reward function.

### 3.1. State Space

**Figure 4:**
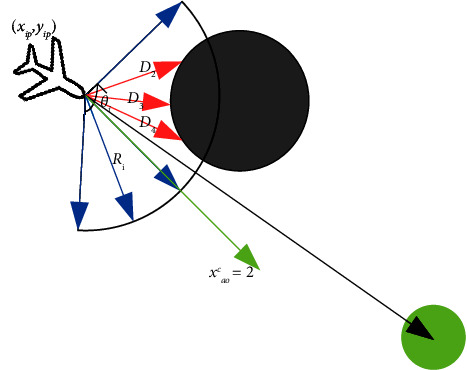
Schematic diagram of UAVs selecting the optimal direction.

The position, speed, and heading of the attack UAV *a* can be characterized as [*x*_*ap*_, *y*_*ap*_, *v*_*a*_, *ψ*_*a*_]. The discretization number *l* of the radar detection range is set to 7, so the detection state can be characterized as *s*_*ao*_ = [*x*_*ao*_^1^, *x*_*ao*_^2^, *x*_*ao*_^3^, *x*_*ao*_^4^, *x*_*ao*_^5^, *x*_*ao*_^6^, *x*_*ao*_^7^]. The attack UAV usually knows the position of the target area in advance. To simplify the input state dimension of the UAV, the position of the target is combined with the radar detection state. As shown in Figure 4, the direction corresponding to the maximum value of state quantity *x*_*ao*_^*i*^, (*i* = 1, ..., *l*) in *s*_*ao*_ (there may be multiple such directions, such as the four blue ray directions in Figure 4) will be determined, and then the direction with the smallest angle with the UAV target line of sight direction will be selected as the optimal heading (such as the green ray direction in the figure) of the attack UAV. The number of this direction is marked as *c*(1 ≤ *c* ≤ 7), and let *x*_*ao*_^*c*^ equals to 2, which means that the attack UAV moves in this direction as much as possible.

In summary, the state of the attack UAV includes the UAV's own position, speed, heading angle, the radar's detection state, and the target's direction. Therefore, the state contains 10 dimensional data in total, which is defined as formula:

(4) $$\begin{array}{c} s_{a}=\left[s_{ap},s_{ad},s_{av},s_{ao}\right]=\left[x_{ap},y_{ap},\psi_{a},v_{a}{,x}_{ao}^{1},x_{ao}^{2},x_{ao}^{3},x_{ao}^{4},x_{ao}^{5},x_{ao}^{6},x_{ao}^{7}\right]\text{.} \end{array}$$

For the defense UAV *d*, the status is similar to the attack UAV, which is defined as formula.

(5) $$\begin{array}{c} s_{d}=\left[s_{dp},s_{dd},a_{dv},s_{do}\right]=\left[x_{dp},y_{dp},\psi_{d},v_{d},x_{do}^{1},x_{do}^{2},x_{do}^{3},x_{do}^{4},x_{do}^{5},x_{do}^{6},x_{do}^{7}\right]\text{.} \end{array}$$

### 3.2. Action Space

It is assumed that the attack UAVs have stronger maneuverability. The control inputs of both UAVs are acceleration and angular velocity, and the action space is shown as formula:

(6) $$\begin{array}{c} A=\left[a_{i},\omega_{i}\right]\left(i=a,d\right)\text{.} \end{array}$$

### 3.3. The Reward Function

Reinforcement learning mostly uses sparse rewards in the field of AI games and has achieved good results [16]. However, the sparse reward cannot make the UAVs to learn efficiently at the beginning of the confrontation task.

Therefore, the reward function of this experiment is set by the combination of guided reward and sparse reward. The design of guided reward *R*_*g*_ is shown in the following formula:

(7) $$\begin{array}{c} \left\{\begin{array}{c} R_{d}=d_{t-1}-d_{t}\text{,}\\ R_{h}=\overset{7}{\underset{n=1}{\sum }}\left(x_{io}^{n}-1\right),\\ R_{v}=\frac{v_{i}}{v_{i\;\max}}\text{,}\\ R_{c}=\left|\psi_{opti}-\psi_{i}\right|\text{,}\left(i=a,d\right), \end{array}\right. \end{array}$$

where *d*_*t*−1_ and *d*_*t*_ represent the relative distance between the UAV and the target at time *t* − 1 and *t*, respectively. *R*_*d*_ represents the variation of relative distance. *R*_*h*_ represents the cumulative value of the UAV radar detection state variable *x*_*io*_^*n*^ relative to 1. *R*_*v*_ represents the reward of the current speed of the UAV. *R*_*c*_ represents the deviation of the current heading *ψ*_*i*_ of the UAV from the optimal heading *ψ*_*opti*_.

(8) $$\begin{array}{c} R_{g}=\alpha_{1}R_{d}+\alpha_{2}R_{h}+\alpha_{3}R_{v}+\alpha_{4}R_{c}\text{.} \end{array}$$

The design of sparse reward *R*_*s*_ is expressed in the following formula:

(9) $$\begin{array}{c} \begin{array}{cc} R_{s}=\left\{\begin{array}{cc} R_{1,} & if\left(x_{ip}\leq x_{\min}\right)or\left(x_{ip}\geq x_{\max}\right)or\left(y_{ip}\leq y_{\min}\right)or\left(y_{ip}\geq y_{\max}\right)\text{,}\\ R_{2}\text{,} & if\;dis\left(s_{ip},s_{io}\right)\leq R_{o}^{k}\left(k=1,L,N_{k\;}\right)\text{,}\\ R_{3,} & if\;dis\left(s_{ip},s_{it}\right)\leq R_{t}orR_{f}\text{,} \end{array}\right. & \left(i=a,d\right) \end{array}\text{.} \end{array}$$

where *R*_1_ represents the penalty for the UAV colliding with the boundary. *R*_*o*_^*k*^ represents the radius of the *k* − th obstacle. *dis*(·) represents the Euclidean distance in two-dimensional space. *R*_2_ represents the penalty for UAV colliding with the obstacle. *R*_*t*_ represents the radius of the target area. *R*_*f*_ represents the attack distance of the defense UAV. *R*_3_ is the reward of the attack UAV to reach the target or the punishment for it being destroyed. The success signal of the defense UAV is that the attack UAV is destroyed.

### 3.4. The DDPG Algorithm

**Figure 5:**
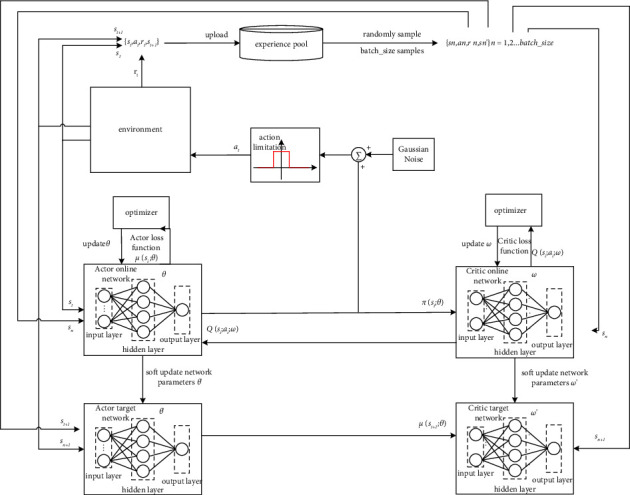
The DDPG algorithm network structure.

The DDPG algorithm is a classic reinforcement learning algorithm based on the actor-critic framework [17]. It is a deterministic policy gradient algorithm referring to the experience playback mechanism and the dual network structure in the DQN algorithm, and it realizes the direct mapping from the continuous state space to the specific high-dimensional action space through the actor network. The network architecture of DDPG is shown in Figure 5.

As shown in Figure 5, the algorithm mainly includes the interactive environment, the experience pool, and the network module of the algorithm. Before the UAV interacts with the environment, it is necessary to determine the number of layers and nodes of the network. We need to initialize the current network parameters randomly and copy the evaluated network parameters to the corresponding target network for the first time. In each step of interaction, the initial state *s*_*t*_ of environmental feedback is taken as the state input of the actor evaluated network, and the action value *μ*(*s*_*t*_; *θ*) of UAV is obtained by the actor network. We need to add Gaussian noise to increase the exploration of the action space on this basis. Due to the limitation of the UAV's angular velocity, the action of the UAV is the combination of Gaussian noise and motion constraints, which is expressed in the following formula:

(10) $$\begin{array}{c} a_{t}=f_{clip}\left(\mu \left(s_{t};\theta \right)+N\right)\text{,} \end{array}$$

where *f*_*clip*_ represents the limitation function of the UAV action, *𝒩* is Gaussian noise, which should obey the formula :

(11) $$\begin{array}{c} N∼N\left(0,\sigma^{2}\right)\text{,} \end{array}$$

where *σ* represents the variance of action noise. The state of the UAVs is determined by the state transition formula (3), and the corresponding reward is obtained according to the reward function. Then, the network training sample [*s*_*t*_, *a*_*t*_, *r*_*t*_, *s*_*t*+1_] is obtained, and we stored it in the experience pool. If the number of samples reaches the requirements for starting training, the parameters of the network are trained according to the method of random sampling. The specific method is to randomly take *m* sets of sample data from the experience pool. {*s*_*n*_, *a*_*n*_, *r*_*n*_, *s*_*n*_′} represents the *n* − th sample. The back propagation algorithm can be used to update the evaluated network parameters.

The loss function *J*(*ω*) of the critic evaluated network is calculated as formula:

(12) $$\begin{array}{c} J\left(\omega \right)=\frac{1}{m}\overset{m}{\underset{n=1}{\sum }}\left({\left(y_{n}-Q\left(s_{n};a_{n};\omega \right)\right)}^{2}\right)\text{,} \end{array}$$

where *ω* represents the parameters of the critic evaluated network, *Q*(*s*_*n*_, *a*_*n*_; *ω*) represents the evaluation value of the critic evaluated network of the current state and the actions performed, and *y*_*n*_ is defined as formula:

(13) $$\begin{array}{c} y_{n}=r_{n}+\gamma Q^{\prime }\left(s_{n}^{\prime };\mu^{\prime }\left(s_{n}^{\prime };\theta^{\prime }\right);\omega^{\prime }\right)\text{,} \end{array}$$

where *r*_*n*_ represents the reward after the UAV performs action *a*_*n*_, *γ* represents the attenuation coefficient of the reward, and *Q*′(*s*_*n*_′; *μ*′(*s*_*n*_′; *θ*′); *ω*′) represents the evaluation value of the critic target network.

The parameter of the critic evaluated network is updated as formula:

(14) $$\begin{array}{c} \omega =\omega -\alpha_{C}\nabla_{\omega }J\left(\omega \right)\text{,} \end{array}$$

where *α*_*C*_ is the learning rate of the critic evaluated network, and ∇_*ω*_*J*(*ω*) is calculated as formula:

(15) $$\begin{array}{c} \nabla_{\omega }J\left(\omega \right)=\frac{1}{m},\overset{m}{\underset{n=1}{\sum }}\left(y_{n}-Q\left(s_{n};a_{n};\omega \right)\right)\nabla_{\omega }Q\left(s;a;\omega \right){|}_{s=s_{n},a=a_{n}}\text{.} \end{array}$$

The parameter updating method of the actor evaluated network:

(16) $$\begin{array}{c} \theta =\theta +\alpha_{A}\nabla_{\theta }J\left(\theta \right)\text{,} \end{array}$$

where *α*_*A*_ is the learning rate of the actor evaluated network. ∇_*θ*_*J*(*θ*) is calculated as formula:

(17) $$\begin{array}{c} \nabla_{\theta }J\left(\theta \right)=\frac{1}{m}\overset{m}{\underset{n=1}{\sum }}\left(\nabla_{a}Q\left(s,a;\omega \right)\left|s=s_{n},a=\mu \left(s_{n};\theta \right)\right.\right.\left.*\nabla_{\theta }\mu \left(s;\theta \right)\left|s=s_{n}\right.\right)\text{.} \end{array}$$

The parameters of the actor target network and the critic target network are updated through a soft update method. Such a slow updating process makes the training process more stable. The process of updating as formula:

(18) $$\begin{array}{c} \left\{\begin{array}{c} \omega^{\prime }\leftarrow \tau \omega +\left(1-\tau \right)\omega^{\prime }\text{,}\\ \theta^{\prime }\leftarrow \tau \theta +\left(1-\tau \right)\theta^{\prime }\text{,} \end{array}\right. \end{array}$$

where *τ* represents the soft update coefficient.

## 4. Confrontation Maneuver Decision-Making Method Based on Transfer Reinforcement Learning

### 4.1. Transfer Learning

It is common that the trained strategies of deep reinforcement learning can only be applied to specific environments. As the complexity of the task increases, it is more difficult for the strategies to apply to new scenarios. Transfer learning is an algorithm that can make full use of the knowledge and experience that could be gained in previous related tasks and applied to new tasks [18]. Transfer learning has a strong ability of model generalization. This idea can also be reflected in daily learning. For example, people use their mother tongue to learn foreign languages. People who are familiar with C++ can quickly learn other programming languages. A solid mathematical foundation is helpful for learning professional courses. All those mentioned previously are based on the previous knowledge to continue learning to solve new problems. Different scenarios or tasks in transfer learning are generally called domains. The domains that have learned experience and knowledge are called source domains, and the domains to be learned are called target domains. The definition of transfer learning is as follows.

Based on the given source domain *D*_*s*_ and source domain task *T*_*s*_, the knowledge *K*_*s*_ learned in the source domain is used to learn *K*_*t*_ in the target domain *D*_*t*_ to complete the task *T*_*t*_ of the target domain.

The idea of transfer learning can also be applied to reinforcement learning. In this paper, the parameter transfer method of transfer learning is used to deal with the scenario of 1vs1 confrontation. The core idea of this method is that the agent learns in a simple task firstly, and if the learned strategy is getting better, the difficulty of the agent's task can be gradually increased. The agent strategies which are suitable for simple tasks will be transferred to more complex tasks to continue learning. This process can reduce the difficulty of exploring complex tasks effectively and avoid the problems caused by sparse rewards successfully.

### 4.2. Confrontation Maneuver Decision-Making Method Based on Transfer Learning

Aiming at the 1vs1 confrontation model established in Section 2, this paper lets the UAV learn in a simple environment firstly and gradually transfer the learned experience to more difficult mission scenarios. In the learning process, when one side's strategy is to be trained, the other side's strategy trained in the previous scenario will be used initially. After the training is completed, the strategy of this training will be used to train the other side. We can use alternate training methods to improve the strategy of the UAVs from the two sides progressively. The specific training process is shown in Table 1.

The pseudocode of the strategy training algorithm for DDPG-based 1vs1 confrontation is shown in Table 2.

## 5. Simulation Experiment

### 5.1. Experimental Environment and Parameter Settings

The experimental software package is PyCharm 2020.1 and Anaconda3. The experimental program is based on the Python language. The settings of the confrontation scenario are shown in Figure 1. This paper uses the standard GUI writing library named Tkinter of Python to build a two-dimensional environment. The neural network is constructed by the PyTorch module, and the version of it is 1.8.1.

The specific parameters of the experimental environment are introduced as shown in Table 3. The obstacles are distributed in each episode randomly, and they are limited in the specific area.

The simulation step Δ*T* is 1s. The PyTorch module is used to build the neural networks of this paper, which all are 3-layer fully connected feedforward neural networks. The number of neurons in each layer of the actor network is [10, 128, 64, 2], and the number of neurons in each layer of the critic network is [12, 128, 64, 1]. The activation function is the ReLU function. To ensure that the action output by the actor network is reasonable, the value output by the final output layer is multiplied by the maximum action limit value by the tanh function. The network parameter optimizer uses the AdamOptimizer module. To reduce the burden of the neural network and speed up the training of the network, the state input of both UAVs will be processed in advance. In this paper, the position coordinates are divided by the maximum boundary length, and the angle is limited to [0,2*π* and divided by 2*π*.

The algorithm training parameter settings are shown in Table 4.

In addition, there are two specific conditions of episode termination in this experiment. One is the number of time steps that the UAV interacts with the environment reaching the maximum number of time steps per episode. The other is that the UAVs collide with obstacles and boundaries or successfully achieve their required targets. For sparse rewards, if the UAV collides with an obstacle or boundary, the rewards *R*_1_ and *R*_2_ are set to −10. If the UAV completes the required task, the reward *R*_3_ is set to 10. For the guided rewards, different reward coefficients *α*_1_, *α*_2_, *α*_3_, and *α*_4_ are set to 0.3, 0.2, 0.2, and 0.3 in formula (8).

### 5.2. Training Result Analysis

The purpose of the reinforcement learning algorithm is to train the agent's strategy to maximize its cumulative reward expectation. The evaluation index of training results can generally be the average reward value of the episode. It is a graph which shows the change of the reward value obtained by the agent training with the number of the episodes. The faster the reward value rises and the more stable and higher the reward value converges, the better the training effect is. This paper uses the average reward of the last 100 episodes as the final average reward value. If there are less than 100 episodes from the beginning of training, only the average reward value of the existing rounds will be used.

**Figure 6:**
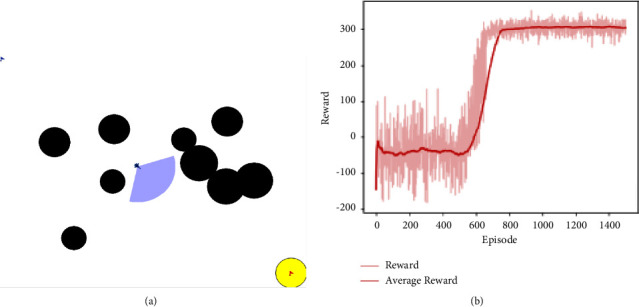
(a) The training environment of step 1 and (b) the average training reward curve.

According to the training steps in Table 2, we can use the strategies trained by the UAVs in the simple task scenarios in step 1 and step 2 to in the scenario of step 3. In step 3, the task difficulty increases gradually, and the transfer and nontransfer methods are used for comparative analysis, respectively. The migration methods are based on the network parameters of 1500 episodes previously trained. The details are as follows:

As shown in Figure 6(a), the offensive UAV has prior information of its starting position and goal position in the environment of step 1, and it is trained to avoid obstacles and boundaries. After 1500 episodes of training, the reward function curve of the attacking UAV is shown in Figure 6(b).

The abscissa of Figure 6(b) represents the number of training episodes, and the ordinate represents the average rewards of the most recent 100 episodes. It can be seen from the figure that the UAV is not clear about what it is going to do at the beginning. It is just exploratory interaction with the environment, and the data of these interactions are extremely useful. After the experience pool is filled (about 520 rounds), as the algorithm begins to train, the reward curve begins to rise gradually, and it starts to show a trend of convergence after 720 episodes with good stability.

**Figure 7:**
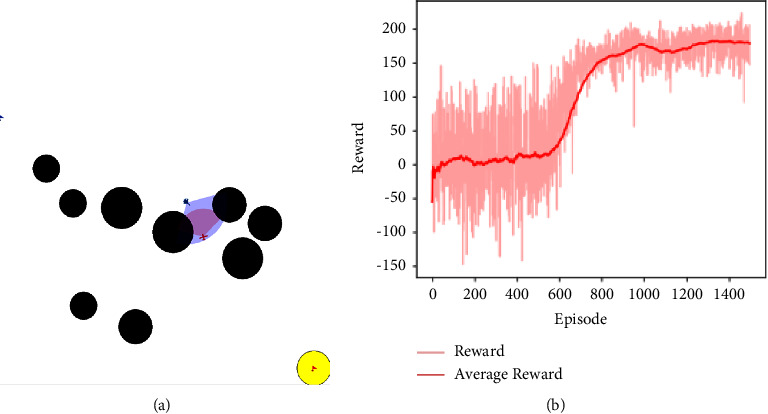
(a) Step 2 training environment and (b) training average reward curve.

As shown in Figure 7(a), in step 2, the defense UAV uses the trained strategy of the attack UAV in step 1 to avoid obstacles and boundaries, and on this basis, the defense UAV is trained to intercept the attack UAV. If the distance from the attack UAV to the target location (yellow) is less than the distance from the defense UAV to the target location, the defense UAV cannot complete the interception and strike mission, it is due to the fact that the maneuverability of the attack UAV is better than the defense UAV. Therefore, the episode will be terminated early, and it means that the attack UAV completes its task successfully and the defense UAV fails to defend.

The abscissa of Figure 7(b) represents the number of training episodes, and the ordinate represents the average rewards of the most recent 100 episodes. It can be seen from the figure that after the experience pool is filled (approximately 580 episodes), the training curve begins to gradually rise and begins to converge around 850 episodes with good stability.

**Figure 8:**
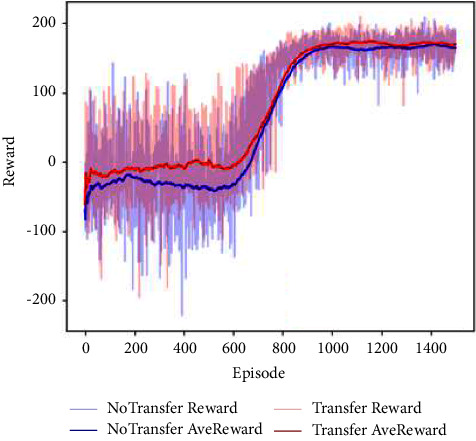
The average reward curve of step 3.

In step 3, the defense UAV used the defensive strategy trained in step 2. It is assumed that the attack UAV can detect the defense UAV by its airborne lidar and take the defense UAV as obstacles to avoid. Then, the attack UAV is trained by the strategy of the attack UAV trained in step 1 and the nontransfer method, respectively. The training results are shown in Figure 8. Similarly, if the distance between the attack UAV and the target position (yellow) is less than the distance between the defense UAV and the target position, the episode will be terminated in advance, and it will be judged that the attack of the attack UAV is successful and the defense of the defense UAV is failed.

The abscissa of Figure 8 represents the number of training episodes, and the ordinate represents the average rewards of the most recent 100 episodes. It can be seen from the figure that both transfer and nontransfer methods can converge within a certain period of time. In contrast, the transfer method has a better round reward value before training and a higher reward value after convergence.

### 5.3. Experiment Result Analysis

In this paper, the training results after 1500 episodes are tested by Monte Carlo for 10000 times. The parameters of the trained actor evaluated network are set in the UAVs.

**Figure 9:**
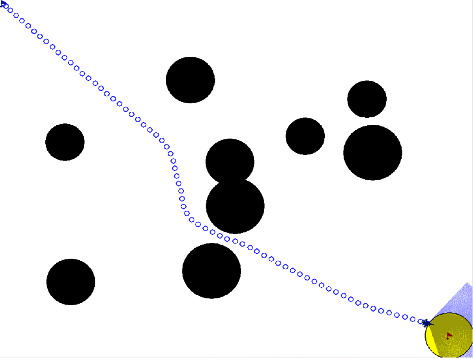
Training results of test step 1 (offensive success).

**Figure 10:**
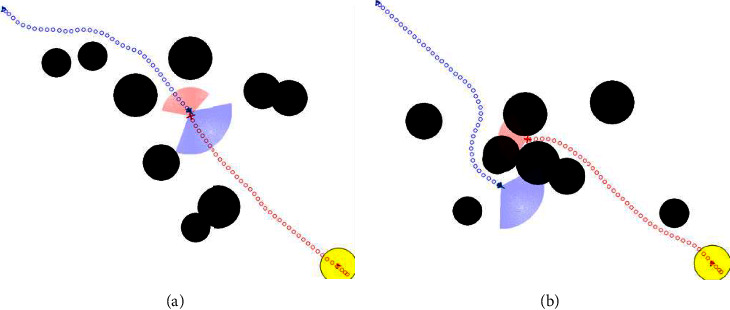
Training results of test step 2 (a) (defense success) and (b) (defense failure).

**Figure 11:**
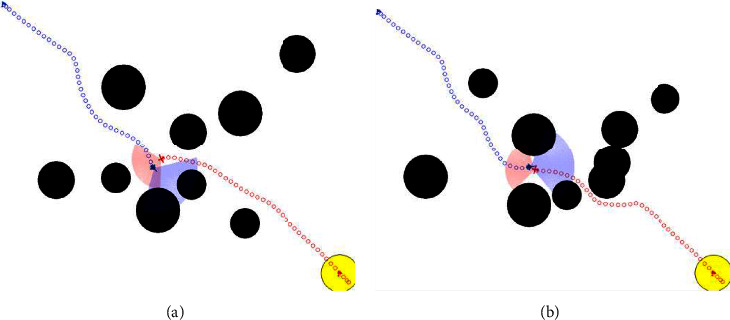
Training results of test step 3 (a) (offense success) and (b) (offense failure).

**Figure 12:**
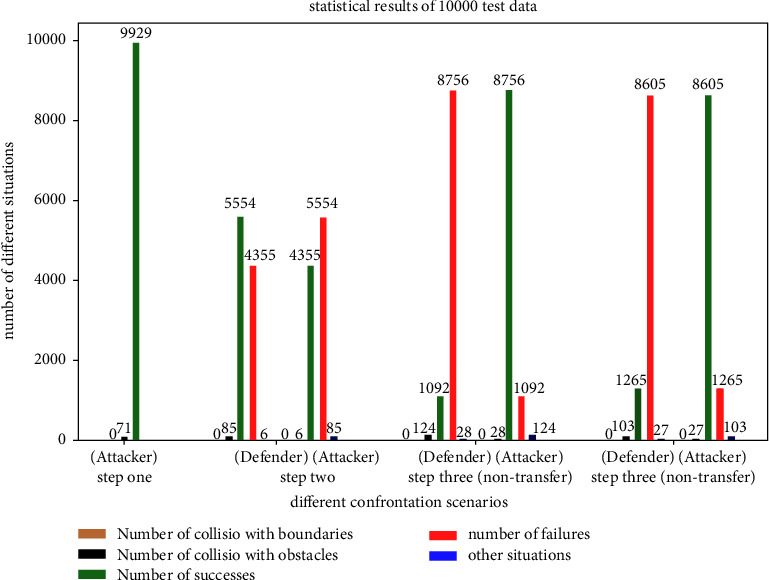
Statistical graph of test data results.

Three different scenarios are tested. The effects of this test are shown in Figures 9–11 (each small circle represents the current position of the UAV at every time step, which is 5 s.). The test result data are shown in Figure 12.

The test results of step 1 show that the attack UAV trained by the presented method can avoid obstacles successfully. The final strategy can achieve stable convergence, and the success rate of avoiding obstacles and reaching the designated area is 99.29%.

The test results of step 2 show the success and failure of the defense UAV, respectively. As shown in Figure 12, both UAVs can avoid obstacles successfully. The defense success rate of the defense UAV is 55.54%. Most of the cases of defense failure are that the two UAVs evade from different sides of the obstacle, so the defense UAV cannot intercept effectively.

The test results of step 3 show the success and failure of the attack UAV, respectively. Compared with the results of the nontransfer method (86.05%), the transfer reinforcement learning method proposed in this paper can increase the offensive success rate (87.56%). Moreover, the results of both sides are greatly improved compared to step 2 (43.55%).

**Figure 13:**
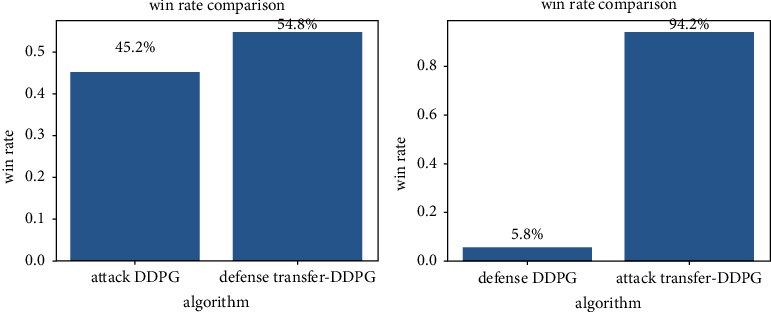
Win rate comparison between the transfer learning algorithm and the DDPG algorithm.

1000 Monte Carlo experiments are conducted between the attackers and defenders trained by the traditional MADDPG algorithm and the attackers and defenders trained by the DDPG algorithm based on transfer learning. The experiment results are shown in Figure 13.

As shown in Figure 13, on the attack side, the winning rate of the transfer learning algorithm is 94.2%, which is significantly higher than MADDPG's 45.2% winning rate, while on the defense side, the winning rate of the transfer learning algorithm is 54.8% which is also significantly higher than MADDPG's 6.8% winning rate. These results demonstrate the effectiveness and superiority of the algorithm proposed in this paper.

## 6. Conclusion

In this paper, reinforcement learning is applied to the UAV confrontation problem, and a 1vs1 confrontation method is designed based on the DDPG algorithm. Based on the model, transfer learning is introduced to train the UAVs. The results show that the proposed method can make training converge faster and can increase the offensive success rate.

Due to its limited mobility, the task success rate of a single defense UAV is not high. Therefore, the next step will continue to study the maneuver decision-making of multiple defense UAVs against a single offensive UAV on the basis of the method proposed in this paper. In the far future, optimizing the framework structure of the algorithm or complicating the environment and adding more UAVs to the scenario will be the development direction.

## Figures and Tables

**Table 1:** The training method of 1vs1 confrontation maneuver decision-making based on transfer learning.

Step	Different scenario requirements from simple to difficult
1	Set that there is only one attack UAV in the battlefield environment and train the UAV to avoid collision with obstacles and boundaries until it can reach the target area
2	Use the strategy of the attack UAV in step 1 and add a defense UAV in the environment. The maneuverability of the defense UAV is not as good as that of the attack UAV. The defense UAV is trained to avoid collision with obstacles and boundaries, and we perform the task of intercepting and attacking the attack UAV
3	Use the strategy of the defense UAV trained in step 2. It is set that the attack UAV can detect the defense UAV in advance. Use the transfer strategy and the nontransfer strategy for training, respectively

**Table 2:** Pseudocode of the 1vs1 countermeasure algorithm based on DDPG transfer learning.

Pseudocode of the 1vs1 countermeasure algorithm based on DDPG
(1) Randomly initialize the parameters *θ* and *ω* of the evaluated network of actor and critic. Initialize experience pool *D* with a capacity of M. The number of initialization batch samples is batch_size. The initial attenuation factor is *γ*. The initial soft update coefficient is *τ*. The initial Gaussian noise variance is noise. The maximum number of initialization rounds is Max_Episode. The maximum number of initialization steps per round is Max_Step
(2) For episode = 1 to Max_Episode do
(3) Obtain the respective state *s*_*t*_ of both sides according to the initial settings of the simulation environment
(4) For *t* = 1 to Max_Step do
(5) Enter *s*_*t*_ as the input of the actor evaluated network to get the UAV's action *a*_*t*_=*f*_*clip*_(*μ*(*s*_*t*_; *θ*)+*𝒩*), where *f*_*clip*_ represents the function of the upper and lower limits of the UAV's restricted action
(6) If there is an enemy UAV, the enemy UAV takes the corresponding confrontation maneuver decision-making according to the description in Table 2and we need to execute action *a*_*ct*_ and update its own state *s*_*ct*_ to *s*_*c*(*t*+1)_
(7) Select the action according to the *ε* − gree dy strategy, that is, training the UAV to randomly select the action within the action range with a certain probability or the action *a*_*t*_ of step 5, then obtain the corresponding reward value *r*_*t*_, and change the environment state to *s*_*t*+1_ at the next moment
(8) Store the sample data [*s*_*t*_, *a*_*t*_, *r*_*t*_, *s*_*t*+1_] of the interaction between the UAV and the environment in the experience pool D
(9) Randomly select batch_size of training sample data [*s*_*n*_, *a*_*n*_, *r*_*n*_, *s*_*n*_′] from experience pool D
(10) Calculate the loss function of the critic evaluated network and update the parameter *ω* of the critic evaluated network through backpropagation to minimize the loss function
(11) Calculate the loss function of the actor evaluated network and update the parameter *θ* of the actor evaluated network through backpropagation loss function
(12) Update the parameters *θ*′ and *ω*′ of the actor and critic target network for every step C
(13) end for
(14) end for

**Table 3:** Design of the training method for 1vs1 confrontation strategies.

Experiment environment parameters	Parameter values
Task area boundary *x*_min_, *x*_max_*y*_min_, *y*_max_	[0, 100] × [0, 80] (km)
Number of obstacles, *N*_*o*_	9
The radius *R*_*o*_^*k*^ and the number of different kinds of obstacles	4 km (3), 5 km (3), 6 km (3)
Area where obstacles appear randomly	[15, 85] × [15, 65] (km)
Radar detection range of attack UAV, *R*_*a*_*∗θ*_*a*_	12 km × 120°
Radar detection range of defense UAV, *R*_*d*_*∗θ*_*d*_	8 km × 120°
Discrete number of detection range, m	7
Maximum speed of attack UAV, *v*_*a* max_	340 m/s
Maximum speed of defense UAV, *v*_*d* max_	300 m/s
The upper limit of the acceleration of the attack UAV, *a*_*a* max_	20 m/s^2^
The upper limit of the acceleration of the defense UAV, *a*_*d* max_	20 m/s^2^
The maximum angular velocity of the attack UAV, *ω*_*a* max_	*π*/15.7
The maximum angular velocity of the defense UAV, *ω*_*d* max_	*π*/22.6
The initial position coordinates and heading angle of the attack UAV	[2.5, 2.5] (km), *π*/4
The initial position coordinates and heading angle of the defense UAV	[97.5, 77.5] (km), 5*π*/4
The center point coordinate (*x*_*tp*_, *y*_*tp*_) and radius *R*_*t*_ of the target area	[95, 75] (km), 5 (km)
The attacking radius of the defense UAV, *R*_*f*_	1 km

**Table 4:** Algorithm training parameter settings.

Parameters	Description	Values
Discount factor, *γ*	Decay factor of cumulative reward	0.95
Inertial update rate, *τ*	Calculate the parameters of the target network by the soft update method	0.01
Experience pool size, M	The sample size of the experience pool, which is the source of training samples	1e5
Number of samples per batch, batch_size	The number of samples used for learning in each batch, randomly chosen from the experience pool	64
Actor network learning rate, *α*_*A*_	Update the parameters of the actor network	1e − 5
Critic network learning rate, *α*_*C*_	Update the parameters of critic network	1e − 4
Exploration rate, *ε*	The exploration rate of the agent's random actions	0.1 ⟶ 0
Action noise, *σ* (variance of the normal distribution)	Action noise variance	3 ⟶ 0
Maximum number of rounds, Max_Episode	Total number of rounds in training	1500
Maximum number of steps per episode, Max_Step	Maximum number of steps in each episode	500
Number of steps between parameter soft update, c	Number of steps between parameter soft update	10

## Data Availability

The data used to support the findings of this study are included within the article.
